# Female urogenital dysfunction following total mesorectal excision for rectal cancer

**DOI:** 10.1186/1477-7819-4-6

**Published:** 2006-01-31

**Authors:** Ian R Daniels, Sheena Woodward, Fiona GM Taylor, Ashraf Raja, Paul Toomey

**Affiliations:** 1 Department of Colorectal Surgery, Epsom General Hospital, Dorking Road, Epsom, UK

## Abstract

**Background:**

The effect of Total Mesorectal Excision (TME) on sexual function in the male is well documented. However, there is little literature in female patients. The aim of this study was to review the pelvic autonomic nervous anatomy in the female and to perform a retrospective audit of urinary and sexual function in women following surgery for rectal cancer where TME had been performed. Urogenital dysfunction was assessed through interview and questionnaire.

**Method:**

Twenty-three questionnaires, eighteen returned, were sent to women with a mean age 65.5 yrs (range 34–86). All had undergone total mesorectal excision for rectal cancer between 1998–2001. Mean follow-up was 18.8 months (range 3–35).

**Results:**

Preoperatively 5/18 (28%) were sexually active, 3/18 (17%) of patients described urinary frequency and nocturia and 7/18 (39%) described symptoms of stress incontinence prior to surgery. Postoperatively all sexually active patients remained active although all described some discomfort with penetration. Two of the patients sexually active described reduced libido secondary to the stoma. Postoperative urinary symptoms developed with 59% reporting the development of nocturia, 18% developed stress incontinence and one patient required a permanent catheter. Of those with symptoms, 80% persisted longer than three months from surgery. Symptoms were predominant in those patients with low rectal cancers, particularly those undergoing abdomino-perineal excision and in those who had previously undergone abdominal hysterectomy.

**Conclusion:**

The treatment of rectal cancer involves surgery to the pelvic floor. Despite nerve preservation this is associated with the development of worsening nocturia and stress incontinence. This is most marked in those patients who had previously undergone a hysterectomy. Further studies are warranted to assess the interaction with previous gynaecological surgery.

## Background

Difficulties with urination and sexual function are common complications after surgery for rectal cancer. In the medical literature male urogenital dysfunction following rectal cancer surgery is becoming increasingly more reported [1]. Assessment in the male consists of the degree of erectile and ejaculatory dysfunction together with assessment of the difficulties of bladder emptying and urinary incontinence. However, there is little literature on female urogenitary dysfunction following rectal cancer surgery.

Two of the main problems when assessing the outcome from surgery for rectal cancer are the development of local recurrence and the morbidity of pelvic autonomic nerve damage. The incidence of sexual dysfunction following conventional rectal cancer surgery, i.e. rectal cancer surgery without consideration of the pelvic autonomic nerves and dissection along the anatomical planes of pelvic fascia are reported to be as high as 94% and the incidence of bladder dysfunction has been documented at 73% [2-4].

The two areas during the dissection where the nerves are at increased risk are the site of the inferior hypogastric plexus, the sympathetic-parasympathetic control site for the bladder during the anterior dissection and the hypogastric nerves are also at risk of damage at the pelvic brim particularly if 'blunt dissection' is used to facilitate a 'flush tie' of the Inferior Mesenteric Artery (IMA). In the female, previous gynaecological surgery may have affected the pelvic autonomic nerve plexus as they pass the cervix and the vaginal fornix laterally to the base of the bladder.

## Patients and methods

Between 1998 and 2001 twenty-three female patients underwent a curative resection for rectal carcinoma. These patients all had tumours within 15 cm of the anal verge and preoperatively had been staged with magnetic resonance imaging and computerised tomography to demonstrate disease limited to the mesorectum (Table 1). The mean age of the patients was 65.5 years (range 34–86). Seven patients underwent an abdomino-perineal excision of the rectum, the remainder an anterior resection (Table 1). The surgical technique was total mesorectal excision with preservation of the pelvic autonomic nerves [5]. Mean follow-up was 18.8 months (range 3–35). To assess urogenital dysfunction a questionnaire was derived from the local current urological assessment proforma and sent to the patients. Eighteen were returned.

**Table 1:** Tumour site, Histology and Operation performed in all patients.

Site above anal verge	Histology (Dukes)	Operation
110 mm	A	AR
40 mm	C	LAR
100 mm	C	LAR
70 mm	B	LAR
150 mm	C	AR
30 mm	A	LAR
40 mm	B	APE
120 mm	B	AR
150 mm	B	AR
50 mm	B	LAR
30 mm	A	APE
10 + 120 mm	A + B	APE
70 mm	A	LAR
40 mm		APE
70 mm	B	LAR
60 mm	C	LAR
60 mm	A	LAR
40 mm	A	APE

(AR: Anterior Resection. LAR: Low anterior resection. APE: Abdomino-perineal Excision)

## Results

Preoperatively 5/18 (28%) of patients were sexually active, 3/18 (17%) of patients described urinary frequency (greater than 6 times per day) and nocturia and 7/18 (39%) described stress incontinence (the leakage of urine with a raised intra-abdominal pressure) prior to surgery. In the sexually active patients there were no symptoms of vaginal dryness or dysparunia. Two patients had severe urinary incontinence. All patients had experienced vaginal childbirth. Five patients had undergone a previous total abdominal hysterectomy and oophrectomy. Three patients were taking hormone replacement therapy.

Postoperatively all sexually active patients remained active although all described some discomfort with penetration. There was no associated vaginal dryness. Two of these patients described reduced libido secondary to the stoma.

**Figure 1:**
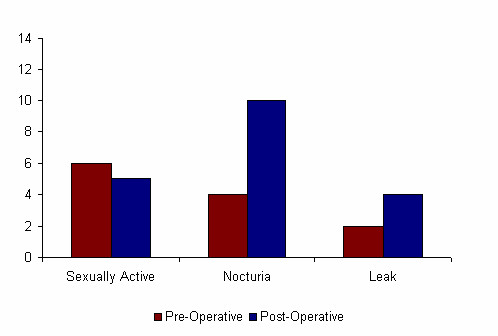
Change of urinary symptoms.

Postoperative urinary symptoms developed with 11/18 (61%) reporting the development of nocturia, 3/15 (20%) developed stress incontinence and one patient required a permanent catheter. All of the patients who had undergone a hysterectomy previously developed nocturia (Figure 1). Of those with symptoms, 80% persisted longer than 3 months from surgery. Symptoms were predominant in those patients with low rectal cancers, particularly those undergoing Abdomino-perineal excision (APE).

## The pelvic autonomic nerves in the female

The pelvic autonomic nerves consist of sympathetic and parasympathetic components. The sympathetic part arises from the ventral roots of L2–L3. After forming a pre-aortic plexus (the superior hypogastric plexus), the sympathetic fibres enter the pelvis as the paired hypogastric nerves, either side of the sacral promontory approximately 10 mm to either side of the midline. The hypogastric nerves continue parallel and medial to the ureter and internal iliac artery and pass in a caudal and lateral direction reaching the pelvic autonomic plexus at the pelvic sidewall where they lie posterior to the peritoneum and directly anterior to the visceral fascia.

The parasympathetic part of the pelvic autonomic nerves arises from the ventral roots of S2–S4 in females. They enter the pelvis through the sacral foramina, posterior to the parietal fascia. The sacral nerves continue to the pelvic autonomic plexus in a caudal and lateral direction. They are covered by the parietal fascia in the first 30 mm of their intra-pelvic path before they cross the retro-rectal space in a double leaf of fascia. Branches are given off medially as the splanchnic nerves forming the parasympathetic supply to the rectum. This is often referred to as the lateral ligament of the rectum.

The inferior hypogastric plexus is a rhomboid shaped plaque of nervous tissue located on the pelvic side wall stretching from an area antero-lateral to the rectum, passing the cervix and vaginal fornix laterally and extending to the lateral vaginal wall and base of the bladder.

The parasympathetic nerves are responsible for increased blood flow to the vagina and vulva, causing vaginal lubrication and swelling of the labia and clitoris. The parasympathetic nerves innervate the detrusor muscle and are therefore necessary for mictuition. The sympathetic nerves are responsible for emission and the rhythmic contractions of the genital ducts and organs during orgasm. The sympathetic nerves inhibit contraction of the detrusor muscle and promote constriction of the bladder neck, ensuring urinary continence. Proprioceptive afferent fibres from stretch receptors in the bladder wall, responsible for the awareness of bladder filling, follow the same pathways as the parasympathetic nerves [5-7].

Disturbances to bladder function may present as various complaints. Damage to the sacral splanchnic nerves may lead to detrusor denervation and decreased sensitivity of the bladder – associated symptoms are difficulty in bladder emptying, overflow incontinence and loss of sensation to fullness of the bladder. Posterior tilting of the bladder, which may occur after an APE, may also cause difficulty in bladder emptying. Urinary tract infection may develop secondary to overflow incontinence. This may be further marked in patients who have undergone a hysterectomy. Loss of sympathetic innervation, which may be the result of damage to the hypogastric nerves, may result in urgency and stress incontinence.

## Discussion

There is little information on female sexual function after rectal cancer surgery. In our study data on post-operative sexual function in women was inconclusive, since a high percentage of the female patients were sexually inactive or indifferent to sexual intercourse before surgery. The number of patients was also small since the female patients did not answer the questions, or only selectively answered the questions regarding sexual health, a problem that has been previously encountered in other studies [8-10]. In patients who are sexually active both decreased libido (reported to range from 25%–83% of patients) and dysparunia (reported to range from 10%–65% of patients) have been seen in our series [2,11-13]. Elderly patients may already experience a declining or absent sexual function before the operation: this may be physiological or related to a higher prevalence of arteriosclerosis, hypertension, diabetes or the use of cardiac and anti-hypertensive medication. Factors other than damage to the pelvic autonomic nerves may influence bladder and sexual dysfunction after rectal cancer surgery. Adjuvant radiotherapy may cause fibrosis of the bladder or vagina affecting vascular permeability.

In patients undergoing APE the perineal resection may also affect the sacral splanchnic nerves causing sexual dysfunction. Distal branches of the pelvic autonomic nerves to the vulva and the clitoris are probably at risk through this approach. Further more the altered pelvic floor anatomy after perineal resection may further influence function. The role of pudendal neuropathy and sphincter damage associated with childbirth trauma has not been assessed. There may also be the effects of previous gynaecological surgery on the pelvic floor. This is particularly important in patients who have undergone a previous hysterectomy where there may have been pre-existing damage to the nerves from the surgery due to their relationship to the cervix and postero-lateral vagina.

## Conclusion

There is little information on female sexual morbidity following TME compared to male. However, we have shown marked urinary dysfunction following surgery despite attempted nerve preservation. This may be associated with previous gynaecological surgery and the effects of childbirth trauma. Further large prospective studies are needed to clearly define the morbidity and thus allow an accurate discussion with the patients when obtaining informed consent for treatment.

## Competing interests

The author(s) declare that they have no competing interests.

## Authors' contributions

**FGM **participated in the design of the study and performed the data collection with **IRD **and **SW**. **IRD and SW **conceived the study, and participated in its design and coordination and helped to draft the manuscript. The patients assessed were under the care of **MAR **and **PT**. All authors read and approved the final manuscript.
